# Supplementary figures and images for: Differential mosquito attraction to humans is associated with skin-derived carboxylic acid levels
Source: Cell. Author manuscript; Available in PMC 2023 Oct 27. (PMC10069481; doi:10.1016/j.cell.2022.09.034)

# A

*Ir76b*<sup>61</sup>

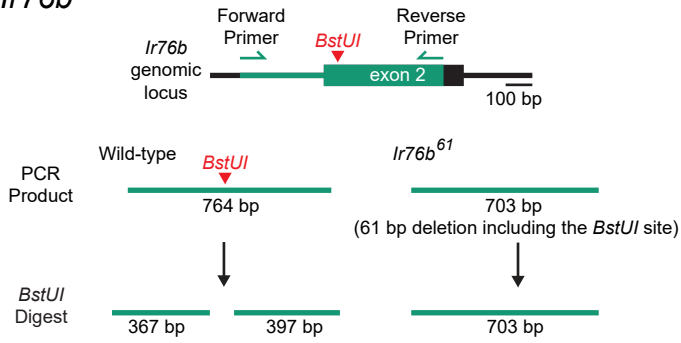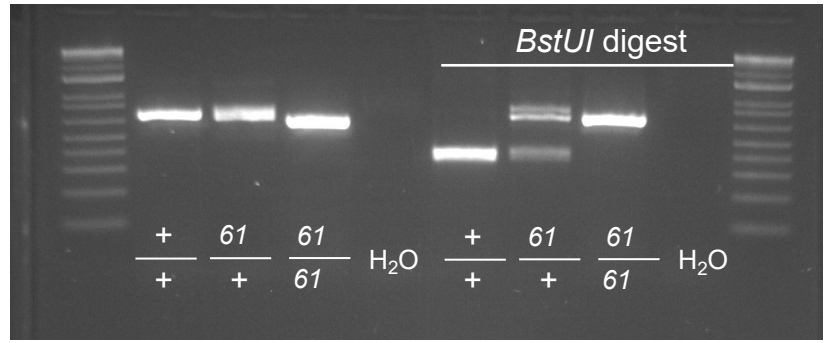

*Ir76b*<sup>32</sup>

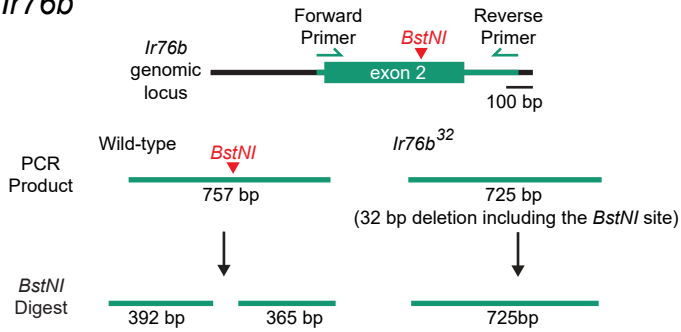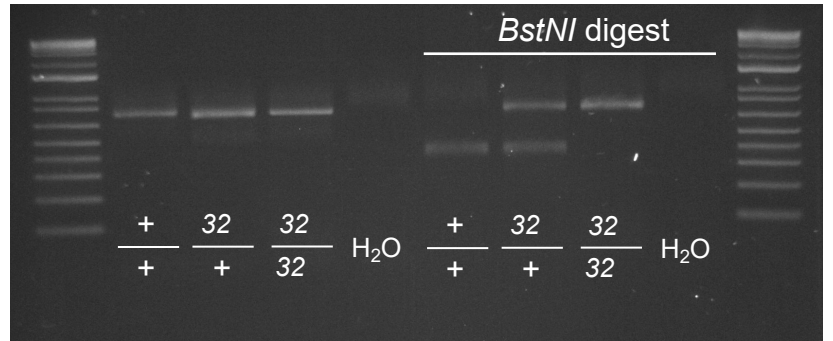

# B

*Ir25a*<sup>BamHI</sup>

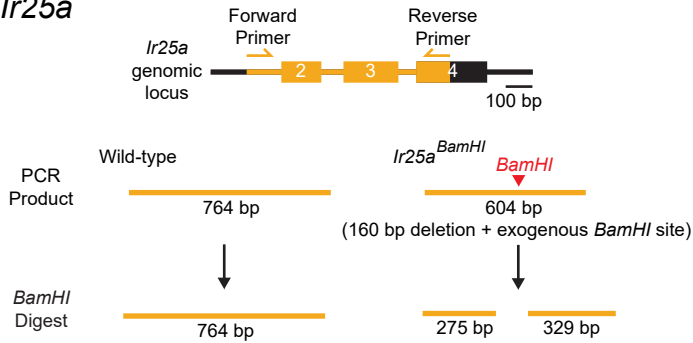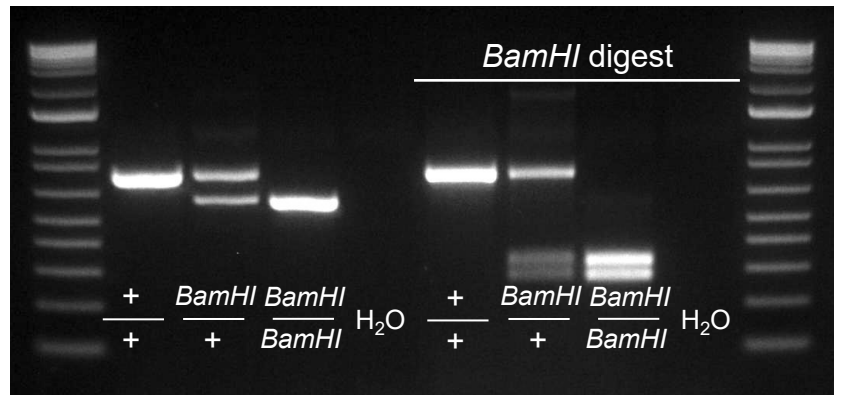

*Ir25a*<sup>19</sup>

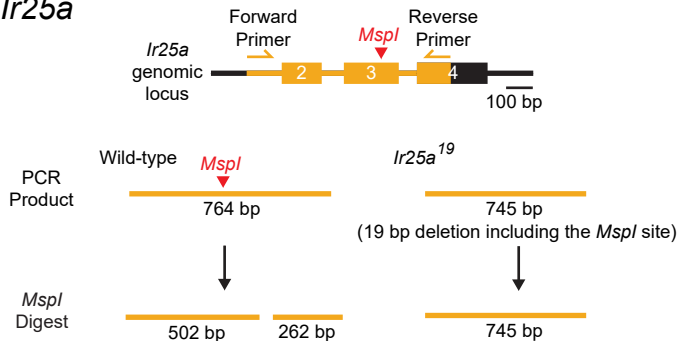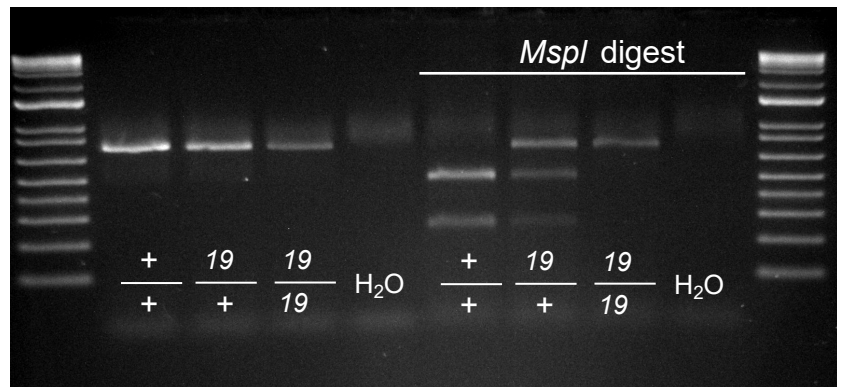

Supplement: 1 — Supplemental Figure S1 - Related to Figure 3 PCR genotyping Ir76b and Ir25a mutant strains (A-B) Genotyping schematics (left) and agarose gel electrophoresis images (right) of PCR fragments before (left) and after (right) restriction with the indicated restriction enzyme to genotype the indicated Ir76b (A) and Ir25a (B) mutants. [file NIHMS1843380-supplement-1.pdf]
