# Supplementary material for: Differential mosquito attraction to humans is associated with skin-derived carboxylic acid levels
Source: Cell. Author manuscript; Available in PMC 2023 Oct 27. (PMC10069481; doi:10.1016/j.cell.2022.09.034)
Supplement: 2 — Supplemental Figure S2 - Related to Figure 5 Additional description and validation of GC/QTOF-MS data (A) Mechanism of pentafluorobenzyl-bromide (PFB-Br) derivatization reaction. (B) Representative extracted ion chromatograms (EICs) for the indicated compounds. (C) Abundance of several carboxylic acids in low attractors (Subjects 19, 28) vs. high attractors (Subjects 31,33). Each dot represents abundance of the indicated compound for one subject in one experiment (median of 4 replicate samples). Nonparametric linear mixed-effects model followed by Benjamini Hochberg FDR correction (p<0.1) was used. Violins labeled by different lowercase letters were significantly different. Among 9 compounds analyzed here, all were verified to be significantly more abundant in the high attractors than low attractors, except tridecanoic acid, which was not significantly different (p=0.126). (D) Representative extracted ion chromatograms (EICs) for three control compounds: two deuterated internal standards and one nylon-derived compound, from the indicated human subjects in Experiment 1.1. (E) Abundance of control compounds shown in (C). Each dot represents the abundance (median of 4 replicate samples) of the indicated compound for one subject in 1 of 4 experiments: Experiments 1.1–1.4). [file NIHMS1843380-supplement-2.pdf]

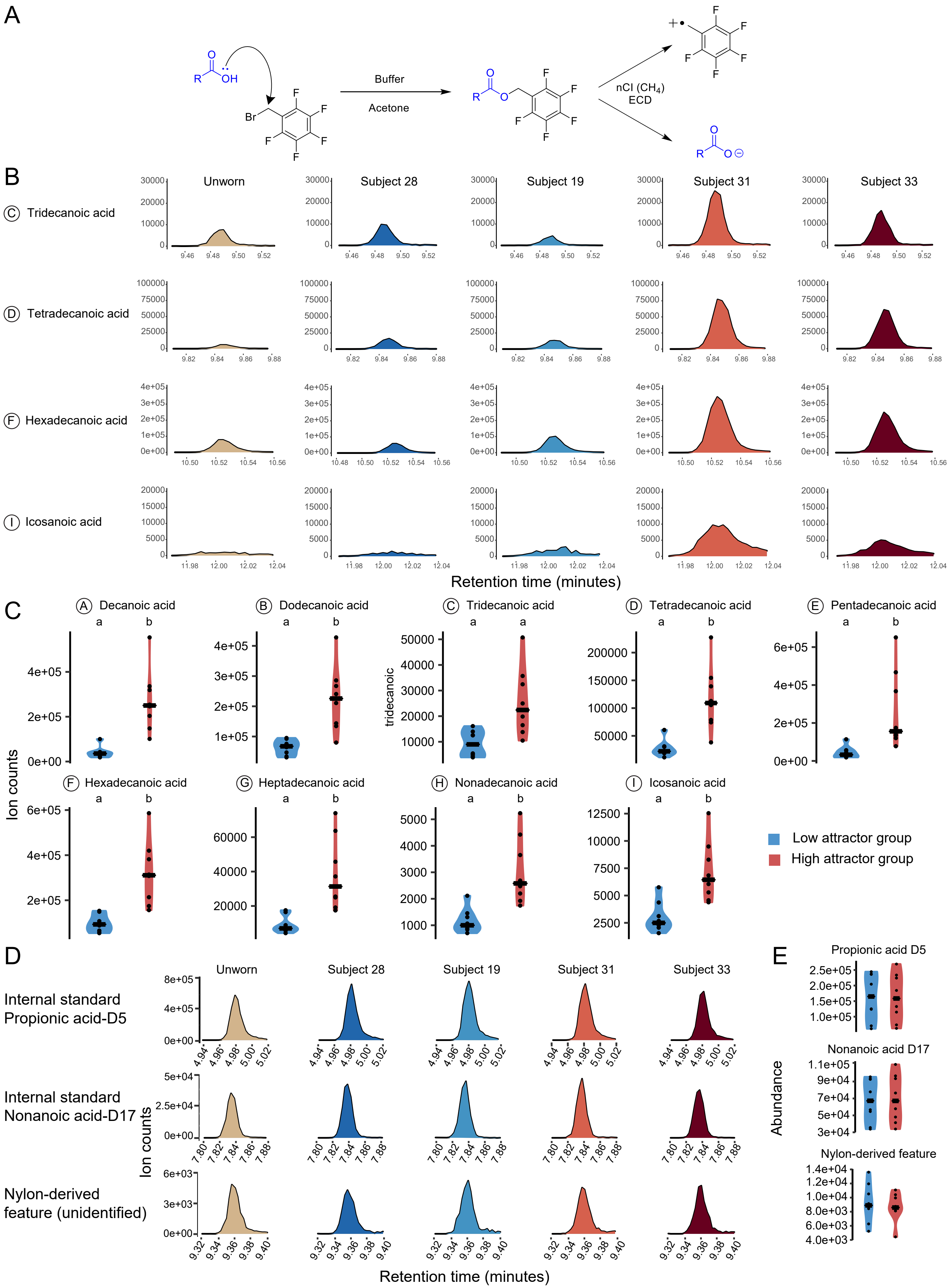

A
Buffer
Acetone
nCI (CH_4)
ECD
B
Unworn
Subject 28
Subject 19
Subject 31
Subject 33
Ⓒ Tridecanoic acid
Ⓓ Tetradecanoic acid
Ⓕ Hexadecanoic acid
Ⓘ Icosanoic acid
Retention time (minutes)
C
Ⓐ Decanoic acid
Ⓑ Dodecanoic acid
Ⓒ Tridecanoic acid
Ⓓ Tetradecanoic acid
Ⓔ Pentadecanoic acid
Ⓕ Hexadecanoic acid
Ⓖ Heptadecanoic acid
Ⓗ Nonadecanoic acid
Ⓘ Icosanoic acid
Ion counts
tridecanoic
Low attractor group
High attractor group
D
Unworn
Subject 28
Subject 19
Subject 31
Subject 33
Internal standard
Propionic acid-D5
Internal standard
Nonanoic acid-D17
Nylon-derived
feature (unidentified)
Ion counts
Retention time (minutes)
E
Propionic acid D5
Nonanoic acid D17
Nylon-derived feature
Abundance
