# Supplementary material for: Differential mosquito attraction to humans is associated with skin-derived carboxylic acid levels
Source: Cell. Author manuscript; Available in PMC 2023 Oct 27. (PMC10069481; doi:10.1016/j.cell.2022.09.034)
Supplement: 4 — Supplemental Figure S4 - Related to Figure 5 and Figure 6 Intra-individual stability of skin chemistry A) Timeline of behavior (green blocks) and GC/QTOF-MS experiments (purple blocks), relative to one another. 64 human volunteers participated in this study, and nylons from 18 of these were analyzed by GC/QTOF-MS. Nylons from four of these subjects (Subjects 19, 28, 31, 33) were repeatedly tested behaviorally over a three-year period and analyzed using GC/QTOF-MS in 2 sets of 4 replicate experiments (Experiments 1.1–1.4, Experiments 2.1–2.4) that were conducted 1 year apart. (B) Heatmaps depicting quantified abundance of carboxylic acids with 10–20 carbons, averaged across 4 replicate samples per experiment, in 4 subjects. Each heatmap represents one of 8 independent experiments. Experiments 1.1–1.4 were conducted about a year before Experiments 2.1–2.4. (C) Heatmaps depicting quantified abundance of carboxylic acids with 10–20 carbons, averaged across 5 replicate samples per experiment, in 18 subjects from the validation cohort. Each heatmap represents one of 4 independent experiments, conducted about a week apart. [file NIHMS1843380-supplement-4.pdf]

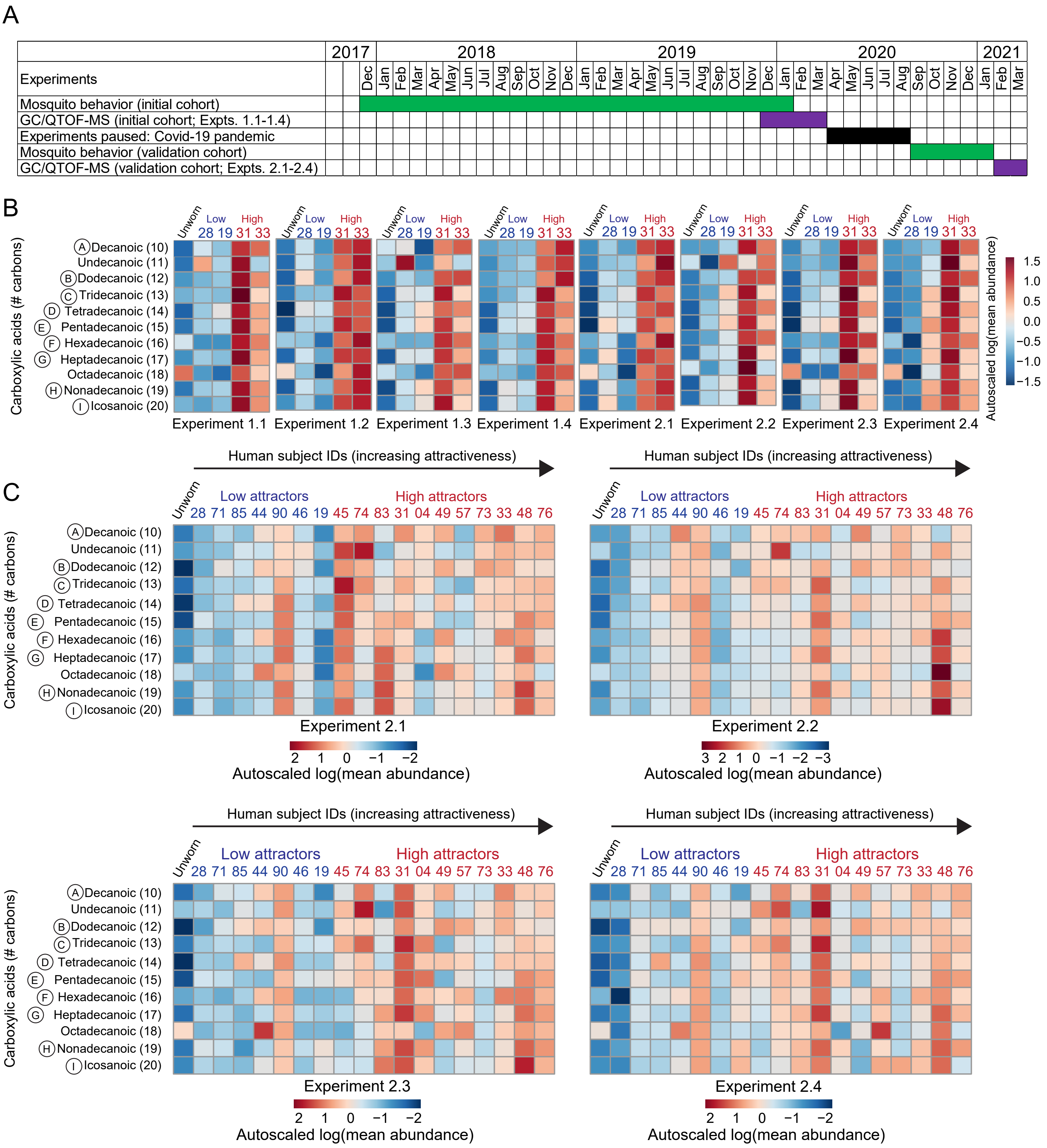
A
2017
2018
2019
2020
2021
Experiments
Dec
Jan
Feb
Mar
Apr
May
Jun
Jul
Aug
Sep
Oct
Nov
Dec
Mosquito behavior (initial cohort)
GC/QTOF-MS (initial cohort; Expts. 1.1-1.4)
Experiments paused: Covid-19 pandemic
Mosquito behavior (validation cohort)
GC/QTOF-MS (validation cohort; Expts. 2.1-2.4)
B
Unworn
Low
High
28 19 31 33
Carboxylic acids (# carbons)
A Decanoic (10)
Undecanoic (11)
B Dodecanoic (12)
C Tridecanoic (13)
D Tetradecanoic (14)
E Pentadecanoic (15)
F Hexadecanoic (16)
G Heptadecanoic (17)
Octadecanoic (18)
H Nonadecanoic (19)
I Icosanoic (20)
Experiment 1.1
Experiment 1.2
Experiment 1.3
Experiment 1.4
Experiment 2.1
Experiment 2.2
Experiment 2.3
Experiment 2.4
Autoscaled log(mean abundance)
1.5
1.0
0.5
0.0
−0.5
−1.0
−1.5
C
Human subject IDs (increasing attractiveness)
Unworn
Low attractors
High attractors
28 71 85 44 90 46 19 45 74 83 31 04 49 57 73 33 48 76
Experiment 2.1
2 1 0 −1 −2
Autoscaled log(mean abundance)
Experiment 2.2
3 2 1 0 −1 −2 −3
Autoscaled log(mean abundance)
Experiment 2.3
2 1 0 −1 −2
Autoscaled log(mean abundance)
Experiment 2.4
2 1 0 −1 −2
Autoscaled log(mean abundance)
