# Supplementary material for: Differential mosquito attraction to humans is associated with skin-derived carboxylic acid levels
Source: Cell. Author manuscript; Available in PMC 2023 Oct 27. (PMC10069481; doi:10.1016/j.cell.2022.09.034)
Supplement: 5 [file NIHMS1843380-supplement-5.pdf]

Supplementary Table S1 - Related to STAR Methods

Untargeted workflow for GC/QTOF-MS

Description of analysis step	# features at each step (Experiments 1.1-1.4)	# features at each step (Experiments 2.1-2.4)
1. Feature finding performed on all worn nylon samples (from 4 experiments) – using Agilent Unknowns Analysis software – Sure Mass deconvolution	~133,000	~300,000
2. Initial feature deduplication (R code "step 2")	1,494	1,925
3. Targeted analysis of deduplicated feature list in all samples (Agilent Qualitative Analysis 10)	1,494	1,925
4. Using R code "step 4": replaced zero values with NA, remove features present in <10% of all samples, impute missing values with 1/2 the lowest value for that feature, perform Log2 transformation	Not determined	Not determined
5. Imported data back into Agilent Mass Profiler Professional software: baselining: "none", scaling: "none", and then use a fold change filter to select high quality features in each experiment<br>a. found in ≥50% of samples in ≥1 subject group<br>b. coefficient of variation ≤40% in ≥1 subject group<br>c. 2-fold upregulated (FDR< 0.05) in ≥1 subject group vs. unworn and vs. solvent control group	Exp. 1.1: 404<br>Exp: 1.2: 376<br>Exp: 1.3: 345<br>Exp. 1.4: 378<br><br>(Note: 204 features were found in 4 of 4 experiments)	Exp. 2.1: 633<br>Exp: 2.2: 619<br>Exp: 2.3: 604<br>Exp. 2.4: 597<br><br>(Note:161 features were found in 4 of 4 experiments)
6. Filter on "hits": features differentially abundant in high attractor vs low attractor subject: ≥1.5-fold change (FDR<0.1) (Agilent Mass Profiler Professional – Volcano plot)<br>• Note: In Exp. 1.1-1.4: 2 high attractor subjects: 31 & 33 were compared 2 low attractor Subjects 19 & 28. (Subject 24 was not available for GC/MS analysis)<br>Note: In Exp. 2.1-2.4: 7 low attractor subjects were compared to 11 high attractor subjects	Exp. 1.1: 210<br>Exp. 1.2: 219<br>Exp: 1.3: 147<br>Exp. 1.4: 240<br><br>(Figure 5D shows Experiment 1.1)	Exp. 2.1: 130<br>Exp. 2.2: 106<br>Exp: 2.3: 100<br>Exp. 2.4:105<br><br>(Figure 6D shows Experiment 2.3)
7. Filter on hits found in 4 of 4 experiments (Agilent Mass Profiler Professional – Venn diagram)	93	23
8. Manually remove redundant features from CSV file in Microsoft Excel	51 (Figure 5E,<br>more info at DOI 10.5281/zenodo.5822538)	13 (Figure 6E, more info at DOI 10.5281/zenodo.5822538)
9. Formula prediction (Agilent Qualitative Analysis 10)	42 (more info at DOI 10.5281/zenodo.5822538)	5 (more info at DOI 10.5281/zenodo.5822538)
10. Positive identification of hit compounds (matched to known standard)	9	3
